# Extracorporeal Membrane Oxygenation (ECMO) Support in Special Patient Populations—The Bidirectional Glenn and Fontan Circulations

**DOI:** 10.3389/fped.2018.00299

**Published:** 2018-10-17

**Authors:** Matthew K. Bacon, Seth B. Gray, Steven M. Schwartz, David S. Cooper

**Affiliations:** ^1^The Heart Institute, Cincinnati Children's Hospital Medical Center, Cincinnati, OH, United States; ^2^Departments of Critical Care Medicine and Pediatrics, The Hospital for Sick Children and the University of Toronto, Toronto, ON, Canada

**Keywords:** ECMO, glenn, fontan, single ventricle, cardiac intensive care, outcomes, congenital heart disease

## Abstract

Extracorporeal membrane oxygenation (ECMO) is a support modality used within the pediatric cardiac ICU population as a bridge to recovery or decision in the setting of acute myocardial decompensation, support for combined cardiopulmonary failure or in the setting of refractory cardiopulmonary arrest. Patients with univentricular physiology are at particular risk for decompensation requiring ECMO support. This review will focus upon current evidence and techniques for ECMO support of single ventricle patients who have undergone a stage II bidirectional Glenn procedure or the stage III Fontan procedure.

## Introduction

Extracorporeal membrane oxygenation (ECMO) is a support modality used within the pediatric cardiac ICU population as a bridge to recovery or decision in the setting of acute myocardial decompensation, support for combined cardiopulmonary failure or in the setting of refractory cardiopulmonary arrest. Common post-operative indications for the use of ECMO in children with congenital heart disease include failure to separate from cardiopulmonary bypass, refractory low cardiac output syndrome, hemodynamically unstable arrhythmias, or extracorporeal cardiopulmonary resuscitation (eCPR). This population is a widely heterogenous group of patients with a variety of independent risk factors and technical difficulties when it comes to extracorporeal support. The use of ECMO for support in the pediatric cardiac population increased each year up until 2014, but since then has remained somewhat stable (1).

This review will focus upon current evidence and techniques for ECMO support of single ventricle patients who have undergone a stage II bidirectional Glenn procedure or the stage III Fontan procedure. We will not focus on stage I single ventricle patients, which make up the majority of single ventricle patients supported with ECMO (2, 3).

## ECMO in patients following the bidirectional glenn procedure

Creation of a superior cavopulmonary anastomosis (bidirectional Glenn procedure) is the second stage of operative palliation for patients with single ventricle anatomy. While outcomes for the Glenn procedure are excellent in the current era, patients are still at risk for decompensation and may require ECMO support. The anatomy of the Glenn circulation and resultant passive pulmonary blood flow pose multiple challenges to cannulation, as well as initiation and maintenance of adequate support.

From a historical standpoint, data regarding ECMO support for Glenn physiology has suggested significant morbidity, poor survival, and this particular anatomy has even been considered a relative contraindication to ECMO (4). Nevertheless, several case reports exist regarding use of ECMO within this patient population. A report detailing peripheral venoarterial (VA) ECMO in a patient with Glenn physiology was published in 2002 in which the authors describe a patient who developed cardiorespiratory failure secondary to respiratory syncytial virus (RSV) infection (5). The patient was successfully supported for 6 days prior to decannulation and ultimately demonstrated adequate Glenn hemodynamics with only minor neurologic sequela. Additionally, another case report in 2010 described the successful use of venovenous (VV) ECMO in a patient with severe hypoxia secondary to isolated pulmonary venous desaturation after the Glenn procedure (6). The patient was successfully decannulated following seven days of support and repeat cardiac catheterization demonstrated adequate hemodynamics. In a larger single center series published in 2004, six patients with Glenn physiology who underwent ECMO support were retrospectively evaluated (7). The authors noted that of the 6 who were cannulated onto ECMO, a total of 3 patients were able to be decannulated. However, all 3 suffered significant neurologic and end-organ injury, with only one patient out of the original 6 ultimately surviving to hospital discharge after undergoing heart transplant. Similar outcomes were confirmed by Alsoufi et al. in a retrospective case series looking at ECMO use in a single center (8). In this series of 100 patients, 31 had single ventricle physiology, 4 of which had Glenn physiology. The authors found that outcomes in the single ventricle group differed based on anatomy and surgical procedure, with patients following the Norwood operation and those following a BT shunt having the highest survival to discharge. Notably, none of the four patients with Glenn physiology who were supported on ECMO ultimately survived to hospital discharge. These studies demonstrate the paucity of data and overall poor outcomes regarding the use of ECMO within this patient population.

While historical data focused on case reports and small series of patients, the current era has turned to databases for outcome data in single ventricle patients supported with ECMO. Using the Society of Thoracic Surgeons (STS) Congenital Heart Surgery database, Mascio et al. evaluated the perioperative use of mechanical circulatory support (MCS) in pediatric heart surgery (9). In this study, the post-operative use of MCS after the bidirectional Glenn was rare (0.8%) but with significant mortality (56% in those treated with MCS compared to 1.6% not requiring MCS). In perhaps the largest study to date, Jolley et al. utilized the Extracorporeal Life Support Organization registry database to evaluate ECMO use in patients after the Glenn operation (10). In this study, 103 infants with Glenn physiology underwent ECMO cannulation, with the vast majority (96%) supported with VA ECMO. The most common indications for ECMO cannulation included cardiac (87%), followed by eCPR (9%), and respiratory (4%). Of the original 103 who were cannulated onto ECMO, 68 were decannulated and 42 ultimately survived to hospital discharge. Survival to hospital discharge for patients with Glenn physiology was lower compared to those treated with cardiac ECMO for all other causes (41 vs. 49%, respectively). However, Glenn survival to discharge after ECMO (41%) was notably higher than for stage 1 palliation (31%) and stage 3 palliation (35%). The authors concluded that while mortality does remain high for this patient population, it is lower than historical studies originally demonstrated and remains reasonable to consider in certain situations.

Patients with Glenn physiology pose unique challenges to successful ECMO support as evidenced by the current outcome data. One of the key components to successful ECMO initiation is establishing adequate venous drainage. The Glenn procedure results in the spatial separation of the superior and inferior caval circulations and thus careful consideration must be given to cannulation strategy. Knowledge of venous anatomy including right, left or bilateral SVCs as well as vessel patency is imperative. Additionally, careful consideration of the etiology of hemodynamic compromise requiring ECMO cannulation should be undertaken. In patients with isolated impaired pulmonary venous oxygenation deficit and without elevated pulmonary vascular resistance or Glenn obstruction, adequate venous decompression may be achieved with isolated cannulation of the inferior caval circulation (6). However, the more likely scenario for hemodynamic compromise in a patient with Glenn physiology will involve elevated Glenn pressure secondary to a variety of etiologies (Glenn circuit obstruction, elevated pulmonary vascular resistance, pulmonary vein stenosis, restriction at the atrial septum, elevated ventricular end-diastolic pressure, depressed ventricular function, etc.) and therefore it may not be possible to achieve adequate venous drainage via a single inflow canula in the IVC. In this scenario, an additional inflow canula placed in the SVC/Glenn circulation is necessary to achieve adequate Glenn (and therefore cerebral) venous decompression.

After obtaining adequate venous cannulation, care must be taken to ensure adequate venous decompression. As previously discussed, patients with failing Glenn physiology have elevated venous pressure that limits pulmonary blood flow and oxygenation as well as effective cerebral venous drainage. High cerebral venous pressures in combination with low systemic blood pressure results in decreased cerebral perfusion pressure and can contribute to neurologic injury, especially in the setting of cardiopulmonary resuscitation. In light of this, certain centers have advocated for cannulation of the SVC/Glenn circulation prior to IVC cannulation; however, no study has confirmed whether sequence of venous cannulation ultimately affects neurologic outcomes (7). A recognized gap in the literature is a detailed account of the most successful cannulation strategies in survivors.

Once adequate venous decompression is achieved, careful consideration must be given to ECMO management to ensure adequate cardiac output and end-organ function. It is imperative to consider the possibility of residual cardiac lesions, obstruction within the Glenn circulation, atrial level restriction, atrioventricular (AV) valve function, or coronary anomalies when evaluating for potentially reversible etiologies of the instigating event necessitating ECMO support. Atrioventricular valve regurgitation is common in failing Glenn physiology, which can result in decreased antegrade cardiac output. Persistent acidosis secondary to inadequate ECMO support and the use of positive pressure ventilation can also decrease pulmonary blood flow, thus further increasing Glenn pressures and raising cerebral venous pressure. Thus, patients who initially only require inferior vena caval cannulation must be monitored carefully for the need to add superior venous drainage. When ECMO is established one can expect to achieve adequate cardiac output with standard flows for age given that patients with Glenn physiology have a Qp:Qs ratio of <1. However, to assure adequate cavopulmonary drainage one might choose to flow higher than normal to guarantee that the ventricular end diastolic pressure remains low.

Similar to the use of ECMO for other cardiac indications, several ECMO complications after the Glenn procedure are associated with decreased survival. Renal failure while on ECMO has been repeatedly demonstrated to be an independent risk factor for mortality during hospitalization (2, 10). Jolley et al. also found that neurologic injury, including seizures, hemorrhage, and embolic stroke, was high in this patient population (10). In terms of survival after eCPR in children with heart disease, renal injury, neurologic injury, and persistent acidosis during ECMO support were all associated with decreased survival to hospital discharge (11). Interestingly, analysis of the National Pediatric Cardiology—Quality Improvement Collaborative data demonstrated that use of ECMO is not a significant predictor of prolonged hospital length of stay after the Glenn operation, although notably this may be due to insufficient sample size (12).

Contemporary data has reported increased use of ECMO in patients with single ventricle physiology over the past decade; however, mortality has remained relatively constant (1, 2). While historical data has demonstrated poor outcomes for bidirectional Glenn patients supported with ECMO, more recent data has suggested that outcomes in the current era may be better than previously reported. Significant morbidity in terms of renal failure and neurologic sequela remain common in patients with Glenn physiology supported on ECMO, and additional studies are needed to understand the optimal management within this patient population.

## ECMO in patients following the fontan procedure

The univentricular patient who has undergone Fontan palliation is a unique and often difficult patient to support with ECMO as well. Unfortunately, in the last 5–10 years there has been a paucity of research and advances when it comes to optimal ECMO support of these patients. Our evidence is limited largely to case reports and larger case series that define outcome data. Retrospective database reviews, although the best we have for this population, fall short by generalizing this quite heterogeneous patient population.

The Fontan operation places the pulmonary and systemic circulations in series, with venous return delivered passively to the lungs followed by the systemic atrium. It is the standard third stage of palliation in single ventricle physiology. The term “failing Fontan” has been used quite broadly to describe the heterogenous group of patients who have developed impaired hemodynamics, either acutely or chronically (13–15). The reality is that failure can result from many underlying issues including arrhythmia, anatomic obstruction to flow, pulmonary vascular remodeling, atrioventricular valve dysfunction, univentricular diastolic dysfunction and chronic underfilling, and/or univentricular systolic dysfunction (16). As one might imagine, the outcome of extracorporeal support largely depends upon the underlying physiology and mechanism for “Fontan failure.”

Supporting Fontan patients on ECMO is functionally feasible but carries high morbidity and mortality (7, 17). In the largest retrospective series to date of 230 patients, Fontan patient survival off ECMO to discharge was 35% (17). During this same period, the overall survival for all cardiac patients on ECMO was 47%. A previously published single center retrospective study published survival as high as 50% post Fontan procedure in a smaller cohort (7). The authors of the former study reported that certain complications were associated with an increased mortality, specifically bleeding and renal failure. As one would expect, those patients with an acute reversible cause tend to do better.

Patients with Fontan circulation pose unique challenges to ECMO support. Adequate venous drainage must first be established and is important for end organ perfusion and recovery (15). This can be quite difficult to achieve with a single cannula, especially in the setting of peripheral cannulation. Even with an adequately sized cannula, decompressing both the upper and lower venous compartments with a single cannula can be difficult depending upon the anatomy of the cavopulmonary connection. Therefore, some patients have required VA ECMO cannulation where venous drainage cannulas are placed in both the upper and lower venous compartments (7). There has been no survival benefit shown when retrospectively reviewing these cannulation strategies, however the studies and limitation of retrospective review prevent any conclusion to such. When adequate venous drainage is achieved, the systemic output usually must be fully supported with ECMO flow. Decompressing the venous compartment, along with a usually elevated transpulmonary gradient in the setting of decompensation, leads to minimal native cardiac output. This requires the need for high ECMO flow (150–200 mL/kg/min) to both decompress and provide adequate circulatory support. However, in the setting of incomplete decompression and desire to avoid stasis in the Fontan circuit or need to maintain of pulsatile flow, one could choose to use a veno-arterial-venous (VAV) strategy. This allows return of some of the oxygenated blood into the Fontan circuit to promote flow through the circuit and return to the systemic ventricle. In addition to the benefit of stasis prevention, this will also assure that the upper part of the body receives oxygenated blood. The care team must be careful because ECMO flow sufficient to support organ perfusion may increase afterload on the native single ventricle. In combination these factors make myocardial recovery and eventual separation from ECMO quite difficult. This is especially true in the Fontan patient with chronic ventricular dysfunction.

It has been suggested that Fontan patients who are receiving CPR at the time of ECMO cannulation (eCPR) have worse outcomes. However, in retrospective analysis there does not seem to be a difference between the two groups, although non-survivors are more likely to receive CPR during their hospital course (7, 17). This is somewhat surprising given the difficulty of maintaining adequate coronary and cerebral perfusion when performing CPR in the setting of Fontan physiology, regardless of the presence of a fenestration (15, 18). Due to the lack of a subpulmonic pumping/capacitance chamber, compressions, and recoil during standard CPR often result in blood moving back through the venous chamber rather than antegrade through the lungs and into the systemic ventricle.

A successful ECMO course with a Fontan patient likely begins with appropriate patient selection, and early recognition. For this reason, understanding the various clinical manifestations of “Fontan failure” is imperative. There are broadly three stages of failure in a Fontan patient each of which is associated with certain underlying etiologies (19). Early Fontan failure is often marked by anatomic obstruction, suboptimal hemodynamics post procedure, or arrhythmia, most commonly atrial flutter or IART. These patients usually have early acute onset of failure, prior to end organ injury. It has been proposed that these patients are the ones who have the best outcomes with ECMO support. Early post-operative ECMO is more easily accomplished via central cannulation thus mitigating some of the anatomic and physiologic challenges particular to the Fontan circulation, and the potential of a bridge to surgical correction of anatomic issues or Fontan conversion or takedown provides more feasible options for effective treatment (20).

With middle and late phase Fontan failure, there is already existing signs of end organ damage. These patients can present with either preserved or diminished ventricular function. The middle phase failure patients are felt to be good transplant candidates and have been supported successfully with ECMO as a bridge to VAD (21, 22). Late phase failure patients present with extreme end organ failure such as protein losing enteropathy, plastic bronchitis, cirrhosis, or renal failure. Although reports exist of these patients being bridged with ECMO to VAD and ultimate transplantation, use of ECMO in this subgroup caries an even higher risk of mortality (19). The majority of the focus of the mid and late phase Fontan failure patients has shifted toward VAD and other forms of mechanical support such as the total artificial heart (19, 23, 24). This is beyond the scope of this review; however, a high percentage of Fontan failure patients were bridged to VAD with ECMO support indicating that it is possible to support these patients if even for a short time.

It has been noted that approximately 30% of Fontan patients develop failure at 20-year follow up (25, 26). Given the low surgical mortality, the number of patients living with Fontan physiology continues to increase. There is no consensus medical management or drug therapy that prevents Fontan failure at this time (26). Literature supports that these patients can be successfully supported on ECMO, although with high morbidity and mortality. It seems as though careful selection of appropriate patients, as well as optimizing ECMO drainage and flow lead to the best outcomes. Specifically, early acute phase failure Fontan patients seem to be the best candidates with ECMO serving as a bridge to organ recovery or surgical correction or conversion. Middle and late phase Fontan failure patients can be supported on ECMO, but most reports indicate that this should be a short-term bridge to more durable mechanical support.

A unique consideration for support in a Fontan patient, specifically those with mid and late phase failure, is whether they present with preserved (i.e., diastolic dysfunction) or decreased systolic function. In Anderson et al. follow up of Fontan patients (16), they report that 73% of patients have preserved systolic function and 72% of patients have diastolic dysfunction. Obviously, there is overlap in these patients and some present with both systolic and diastolic dysfunction. This has led to several groups exploring the use of a Cavopulmonary Assist Device (CPAD) to improve Fontan flow and increase ventricular preload in the setting of preserved systolic function (27–29). The goal would be to modestly improve and augment cavopulmonary pressure (by 2–5 mmHg), and thus improve ventricular filling and increase cardiac output. *In vitro* and *in silico* models have been developed, however thus far there is no evidence of successful patient support. In the extensive review by Rodefeld, he proposes an approach by which patients could be supported by CPAD alone, VAD alone, or combination thereof depending upon the type of Fontan failure at presentation (27).

## Conclusion

There are times that single ventricle patients who have undergone either bidirectional Glenn Procedure or Fontan procedure can benefit from ECMO support even though such support is associated with high morbidity and mortality. However, if adequate venous drainage is established it is possible to achieve successful mechanical support. On the surface there appears to have been little in the way of advances in ECMO support for Glenn and Fontan patients over the past decade, mortality seems to have improved when comparing contemporary publications to those of a decade ago. The majority of the newest advances cited in the literature seem to focus around other means of mechanical support for these patients, such as ventricular assist devices. One can therefore deduce that mortality associated with these devices may be improved largely because of better planning and patient selection prior to acute decompensation. This seems equally important in successful and optimal support of these patients using ECMO.

## Author contributions

MB and SG: draft of the initial manuscript; SS and DC: review and editing of the manuscript.

### Conflict of interest statement

The authors declare that the research was conducted in the absence of any commercial or financial relationships that could be construed as a potential conflict of interest.
